# Editorial: Hemodynamic parameters and cardiovascular changes

**DOI:** 10.3389/fphys.2024.1538859

**Published:** 2025-01-03

**Authors:** Tania Pereira, Kais Gadhoumi, Ran Xiao

**Affiliations:** ^1^ Faculty of Science and Technology, University of Coimbra, Coimbra, Portugal; ^2^ INESC TEC—Institute for Systems and Computer Engineering, Technology and Science, Porto, Portugal; ^3^ Duke University School of Nursing, Durham, NC, United States; ^4^ Nell Hodgson Woodruff School of Nursing at Emory University, Atlanta, GA, United States

**Keywords:** biomedical signal processing, non-invasive cardiovascular condition assessment, hemodynamic parameters, wearables, photoplethysmography

## 1 Introduction

Photoplethysmography (PPG) is a cost-effective and accessible optical sensing technique that can be easily incorporated into wearable devices for non-invasive and continuous monitoring of physiological and hemodynamic changes such as heart rate, respiratory rate, oxygen saturation, vascular stiffness, and cardiac output. The measurement of these changes over a period of time provides insights into cardiovascular and respiratory health, offering opportunities for just-in-time health information that can help in the diagnostication of elusive conditions such as paroxysmal arrhythmia and in the assessment of risk for cardiovascular and neurological diseases.

Continuous monitoring enables early screening of diseases by providing real-time detection of important physiological changes. This can significantly enhance risk stratification, improve the accuracy of clinical decision-making, and lead to better patient outcomes by enabling a timely and appropriate intervention. To achieve these objectives, advanced techniques are needed for identifying complex and peculiar changes in the PPG signal and extracting relevant hemodynamic parameters. Overcoming challenges such as artifacts that significantly alters the PPG signal morphology is essential for the veracity of findings and the trustworthiness of PPG as a diagnostic tool. Emerging biomedical signal processing, pattern recognition, and artificial intelligence methods contributed to the marked advancement of PPG technology and its applications. There remains however potential for tapping further into the complex nature of PPG signals and extract new and nuanced physiological signatures of health. As new techniques emerge that can overcome technical barriers such as refractory motion artifacts, PPG can lead to novel applications in personalized health monitoring ultimately enabling better chronic disease management and preventative care.

## 2 Main findings of the articles

This Research Topic addresses the key requirements for expanding the use of PPG in both daily life and clinical settings, focusing on developing novel hemodynamic parameters, new methods to extract clinical information, and strategies to deal with noise. Contributions to this Research Topic highlight these advancements, which are summarized below.

Cuff-less estimation of blood pressure (BP) has for long time been challenging and ambitious. Its successful implementation holds the potential to revolutionize health monitoring and patient care by improving accessibility and fostering greater patient compliance. The work by Liu et al. on “*Continuous Blood Pressure Estimation From Electrocardiogram and Photoplethysmogram During Arrhythmias*” proposed a machine learning-based approach for cuff-less and continuous BP estimation using electrocardiogram (ECG) and PPG in episodes of ventricular and supraventricular arrhythmias. Hemodynamic parameter estimation is further challenged in arrhythmia periods due to associated morphological variations of the cardiac cycle. Several indices have been derived from the PPG and used as predictive markers of different health outcomes. The review *“Risk factors and predictive indicators of rupture in cerebral aneurysms”* by Wang and Huang highlighted how the advancement in computational fluid dynamics and machine learning can facilitate the creation of new predictive models for assessing rupture risk in cerebral aneurysms. This is particularly important because the structural response of the aneurysmal wall to hemodynamical factors plays a crucial role in aneurysm stability and risk for rupture. The authors discuss hemodynamical and other risk factors associated with cerebral aneurysms and the potential for new physiological and digital biomarkers and artificial intelligence algorithms to enhance the detection, risk assessment, prognosis, and treatment guidance of cerebral aneurysms.

An important use of PPG is to augment patient care with non-invasive technology at the point of care. The combination of PPG with invasive techniques for precise monitoring of vital signs such as arterial line has become key in critical care monitoring. Beat-to-beat arterial blood pressure variability obtained from the analysis of arterial line recordings has been proposed to uncover new indicators of health status. In their study *“Blood pressure fragmentation as a new measure of blood pressure variability: association with predictors of cardiac surgery outcomes*,*”* Costa et al. proposed a novel and new measure of blood pressure variability called *blood pressure fragmentation*. The measure is based on a dynamic approach to assessing cardiovascular dysregulation. A fragmentation analysis was developed to overcome the limitations of time and frequency domain metrics in heart rate variability analysis. Increased preoperative systolic blood pressure fragmentation was found to be associated with higher risk of morbidity and mortality in cardiac patients.

Xu et al. proposed an all-noninvasive approach to characterizing peripheral arterial stiffness and sympathetic activation. The study of arterial stiffness stressors has multiple implications in the assessment of cardiovascular risks including hypertension and atherosclerosis. In their study entitled *“Noninvasive characterization of peripheral sympathetic activation across sensory stimuli using a peripheral arterial stiffness index”* the authors developed a model of the transient response of the arterial stiffness index using continuous arterial pressure monitor and a pulse oximeter signals to characterize the sympathetic activation. The model is potentially capable of continuously tracking sympathetic activators.

Cicone and Wu proposed in their work *“How Nonlinear-Type Time-Frequency Analysis Can Help in Sensing Instantaneous Heart Rate and Instantaneous Respiratory Rate from Photoplethysmography in a Reliable Way”* a novel algorithm to simultaneously extract instantaneous heart rate and respiratory rate from single-channel PPG signals. The algorithm, called *deppG*, is based on a nonlinear masking technique that promises accurate estimates in real-world scenarios, including physically-intense activities.

In their work *“Robust arterial compliance estimation with Katz’s fractal dimension of photoplethysmography*,*”* Xing et al. used PPG to derive a noise-resistant indicator of arterial compliance. Arterial compliance is an important parameter of the cardiovascular function that characterizes the relationship between changes in arterial blood volume and pressure. It significantly influences cardiovascular function, including cardiac performance and pulse wave velocity. The authors used Katz’s fractal dimension of PPG to estimate arterial compliance continuously and robustly, enabling unobtrusive and wearable assessment of cardiovascular health.

## 3 Perspectives and conclusion

This Research Topic provided multidisciplinary research based on physiological studies of signal processing, focusing on relevant applications of the PPG signal and emphasizing the large spectrum of applications and the importance of this technology in continuous health monitoring. Technical challenges surrounding the use of PPG, particularly in mobile health applications, remain. Efforts to address these challenges can be grouped into three main lines of research and development areas:

### 3.1 Innovative methods for extracting new parameters

The PPG signal contains a wealth of valuable physiological information. There remains a compelling need to innovate and develop novel algorithms and techniques to extract additional clinically relevant hemodynamic and physiological parameters from PPG data. Such advancements could encompass more detailed metrics of cardiovascular health, respiratory function, and even markers of stress or fatigue. By refining these methodologies, the utility of PPG for comprehensive health monitoring can be significantly enhanced.

### 3.2 Strategies to mitigate motion artifacts

The formulation of robust methodologies to mitigate or correct noise-induced artifacts is instrumental in guaranteeing the fidelity of PPG-based evaluations in practical and real-word scenarios.

### 3.3 Exploring AI-driven solutions

AI-driven healthcare innovations enable the identification of nuanced health trajectories and deliver tailored insights derived from individual physiological datasets. Furthermore, sophisticated AI algorithms facilitate the automated extraction of salient parameters, recognize intricate patterns that elude conventional detection methods, and significantly advance predictive health analytics.

Addressing these three foundational pillars of development is essential for realizing the full potential of PPG technology. By surmounting current limitations and enhancing the precision in extracting and interpreting PPG data, this research lays the groundwork for broader, more effective, and reliable applications of PPG in routine health monitoring and clinical practice.

